# Urinary Proteomics in Predicting Heart Transplantation Outcomes (uPROPHET)—Rationale and database description

**DOI:** 10.1371/journal.pone.0184443

**Published:** 2017-09-07

**Authors:** Qi-Fang Huang, Sander Trenson, Zhen-Yu Zhang, Wen-Yi Yang, Lucas Van Aelst, Esther Nkuipou-Kenfack, Fang-Fei Wei, Blerim Mujaj, Lutgarde Thijs, Agnieszka Ciarka, Jerome Zoidakis, Walter Droogné, Antonia Vlahou, Stefan Janssens, Johan Vanhaecke, Johan Van Cleemput, Jan A. Staessen

**Affiliations:** 1 Studies Coordinating Centre, Research Unit Hypertension and Cardiovascular Epidemiology, KU Leuven Department of Cardiovascular Sciences, University of Leuven, Leuven, Belgium; 2 Center for Epidemiological Studies and Clinical Trials and Center for Vascular Evaluations, Shanghai Institute of Hypertension, Shanghai Key Laboratory of Hypertension, Ruijin Hospital, Shanghai Jiaotong University School of Medicine, Shanghai, China; 3 Division of Cardiology, University Hospitals Leuven, Leuven, Belgium; 4 Mosaiques Diagnostics GmbH, Hannover, Germany; 5 Biotechnology Division, Biomedical Research Foundation, Academy of Athens, Athens, Greece; 6 R&D Group VitaK, Maastricht University, Maastricht, The Netherlands; University of Glasgow, UNITED KINGDOM

## Abstract

**Objectives:**

Urinary Proteomics in Predicting Heart Transplantation Outcomes (uPROPHET; NCT03152422) aims: (i) to construct new multidimensional urinary proteomic (UP) classifiers that after heart transplantation (HTx) help in detecting graft vasculopathy, monitoring immune system activity and graft performance, and in adjusting immunosuppression; (ii) to sequence UP peptide fragments and to identify key proteins mediating HTx-related complications; (iii) to validate UP classifiers by demonstrating analogy between UP profiles and tissue proteomic signatures (TP) in diseased explanted hearts, to be compared with normal donor hearts; (iv) and to identify new drug targets. This article describes the uPROPHET database construction, follow-up strategies and baseline characteristics of the HTx patients.

**Methods:**

HTx patients enrolled at the University Hospital Gasthuisberg (Leuven) collected mid-morning urine samples. Cardiac biopsies were obtained at HTx. UP and TP methods and the statistical work flow in pursuit of the research objectives are described in detail in the Data supplement.

**Results:**

Of 352 participants in the UP study (24.4% women), 38.9%, 40.3%, 5.7% and 15.1% had ischemic, dilated, hypertrophic or other cardiomyopathy. The median interval between HTx and first UP assessment (baseline) was 7.8 years. At baseline, mean values were 56.5 years for age, 25.2 kg/m^2^ for body mass index, 142.3/84.8 mm Hg and 124.2/79.8 mm Hg for office and 24-h ambulatory systolic/diastolic pressure, and 58.6 mL/min/1.73 m^2^ for the estimated glomerular filtration rate. Of all patients, 37.2% and 6.5% had a history of mild (grade = 1B) or severe (grade ≥ 2) cellular rejection. Anti-body mediated rejection had occurred in 6.2% patients. The number of follow-up urine samples available for future analyses totals over 950. The TP study currently includes biopsies from 7 healthy donors and 15, 14, and 3 patients with ischemic, dilated, and hypertrophic cardiomyopathy.

**Conclusions:**

uPROPHET constitutes a solid resources for UP and TP research in the field of HTx and has the ambition to lay the foundation for the clinical application of UP in risk stratification in HTx patients.

## Introduction

The prevalence of heart failure (HF) among adults living in developed countries is approximately 2%, amounting to 15 million in the European Union [1] and 5 million in the United States [2] with a 5-year mortality rate in excess of 50% [1,3]. The first heart transplantation (HTx) took place in 1967. The procedure is now the treatment of choice for a highly selected group of terminally ill HF patients with severe symptoms not responding to maximum medical therapy with the goal to prolong survival and improve quality of life [4]. Currently, 5000 HTx procedures are carried out each year worldwide, mainly in Europe and North America. HTx is associated with a nearly 85% 1-year survival rate and 90% freedom from symptoms and activity limitations in survivors at 1 to 3 years after HTx. In spite of this undeniable success, HTx programs keep meeting major challenges in responding to the steadily increasing demands. First, the number of HF patients is growing, due to the aging of populations, improved survival after myocardial infarction, and the protracted course of HF treated with modern medical treatment. Advances in immunosuppression and prevention of infection, coupled with better survival after HTx, led to the liberalization of the selection of potential recipients. This trend explains the ever-enlarging gap between the limited supply of donor hearts and demand. Second, recent advances in mechanical circulatory support, specifically implantable left ventricular (LV) assist devices (LVAD), are providing alternatives not only for patients waiting for HTx (bridge to transplantation), but also for patients who are ineligible for HTx (destination therapy) or who might experience recovery after LV unloading (bridge to recovery). Thus, the availability of LVADs helps patients shortlisted for HTx surviving until a donor heart is available. On the other hand, it adds complexity to the management of HF patients and complicates the decision process that the multidisciplinary transplantation teams have to go through to make optimal use of HTx as a treatment modality [4] to balance the high demand with the limited resources (suitable donor hearts).

Capillary electrophoresis coupled with high-resolution mass spectrometry (CE-MS) enables detection of over 5000 peptide fragments in urine samples. Combined in multidimensional classifiers, the urinary proteomic signatures reproducibly identify subclinical diastolic LV dysfunction [5–7], renal impairment [8–10], acute coronary syndromes [11], and even 5-year adverse cardiovascular and cardiac outcomes [12]. The urinary PROteomics in Predicting HEart Transplantation outcomes (uPROPHET; study registration number, NCT03152422) is a proof-of-concept project sponsored by the European Research Council that should lead to the initial validation and clinical application of profiling of the urinary proteome (UP) in HTx patients with the goal to help choosing treatment modalities with the greatest probability of achieving long-term graft survival with high-quality years added to the patients’ life. Moreover, the UP profile might contribute to detecting graft vasculopathy at an early subclinical stage and to monitoring the activity of the immune system and graft performance after HTx and could therefore be of value in the management of immunosuppression. Additionally, previously established UP classifiers will be further validated by demonstrating analogy between the UP profiles in urine and tissue samples of explanted hearts with cardiomyopathy in comparison with healthy but disregarded donor hearts. Access to tissue proteomic (TP) and UP data in combination with bioinformatics tools will help identifying relevant molecular mechanisms in HF and thus pave the way for novel therapies.

## Methods

### Selection of patients

**Fig 1:**
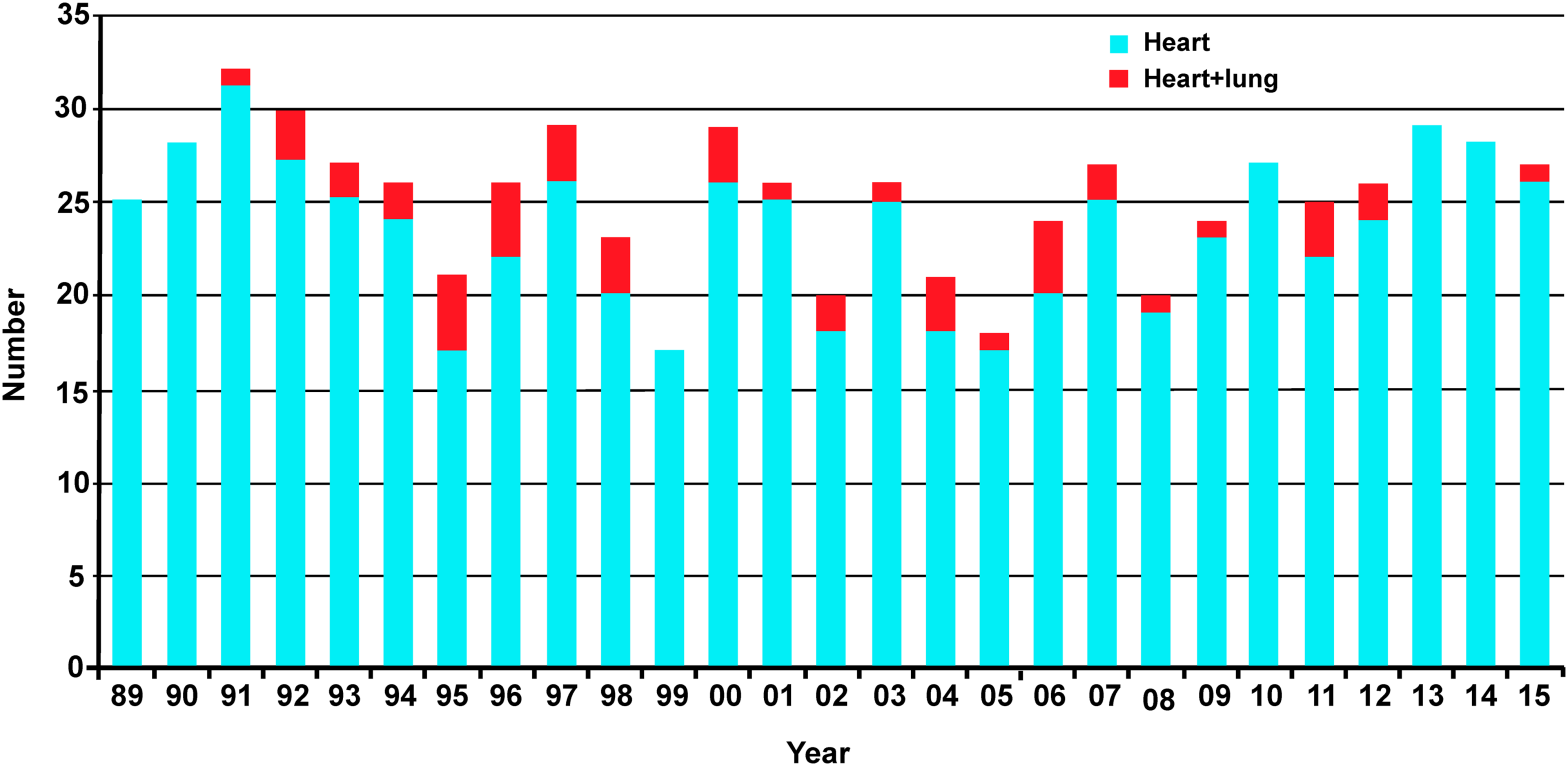
Number of heart transplantations performed at the University Hospitals Leuven from 1989 until 2015.

uPROPHET complies with the Helsinki declaration for research in Humans [13]. The project was approved by the Ethics Committee of the University Hospitals Leuven (approval numbers B322201421186 [S56384] and B322201421045 [S56472]) and passed ethical screening by the European Research Council Executive Agency (ERCEA). HTx recipients provided written informed consent. Recruitment of patients took place at the University Hospital Gasthuisberg in Leuven (2014–2015) in collaboration with the Heart Transplantation Team. After a standardized diagnostic workup performed according to current guidelines [14,15], each year, approximately 30 patients are shortlisted for HTx (Fig 1), of whom most receive LVAD support as bridge to transplantation.

For the TP studies, uPROPHET investigators received anonymized data on donors via the heart transplant coordinator at the University Hospitals Leuven. At the time of writing of this protocol outline, myocardial biopsies were available from 7 healthy donors, whose hearts did not meet current criteria for HTx, mainly because of the advanced age of the donor [16], and were therefore discarded, as well as myocardial tissue obtained from explanted hearts from 15, 14, and 3 patients with ischemic [17,18] dilated [17,19], and hypertrophic [17,20] cardiomyopathy diagnosed and selected for HTx according to current guidelines [15,21]. Hereinafter donor hearts are referred to as controls and diseased hearts as cases.

### Follow-up after heart transplantation

After HTx, right heart catheterization with endomyocardial biopsies (EMB) are routinely scheduled at weekly intervals during the first 6 weeks, every 2 weeks thereafter up to 3 months, followed by every three weeks up to week 18, then monthly up to month 6, and every 2 months for the rest of the first post-transplant year. Between post-transplant years 1 and 2, these assessments take place every 3 months. Afterward, right heart catheterization and EMB are only performed if a rejection is suspected. Following a treated episode of rejection, EMB is generally repeated within 14 days to ensure that immunosuppression is adequate. A coronary angiography is performed 1, 3 and 5 years after HTX and thereafter at 5-year intervals or as clinically indicated. The annual follow-up also includes ambulatory blood pressure monitoring and echocardiography. All potentially relevant information from technical examinations, including invasive hemodynamic testing, echocardiographic data and EMB histopathology were and are being retrieved from the computerized information system of the University Hospitals Leuven.

The chronic antirejection therapy consisted of cyclosporine, azothioprine and a low dose of methylprednisolone before December 1999. Since January 2000, all HTx recipients receive a combination of tacrolimus and mofetil mycophenolate and most go off steroids after the first postoperative year. Unless not tolerated, all patients receive statins, usually pravastatin and those transplanted because of ischemic cardiomyopathy, a low-dose aspirin formulation, if indicated. At each contact, blood levels of anti-rejection drugs are checked to ascertain adherence and adjust the dose of the anti-rejection drugs, if required.

### Definition of endpoints

Patients are being prospectively followed up from the date of the first urine sampling (baseline; 2014–2015) until death or censoring on the date of the last follow-up visit available in the information system of the University Hospitals Leuven. The prospectively defined cardiovascular endpoint includes: sudden death, cardiovascular death, the need for retransplantation, treated acute antibody-mediated or acute cellular rejection, graft vasculopathy, hospitalization for HF and other coronary events. Severe graft vasculopathy includes angiographically documented coronary lesions resulting in a decreased systolic LV performance, admission for an ST-elevation myocardial infarction (STEMI) or non-STEMI infarction, or the need for percutaneous or surgical coronary revascularization. The composite cardiovascular endpoints include all foregoing endpoints as well as transient ischemic attack, defined as a neurological deficit lasting for less than 24 hours, stroke, pulmonary emboli and peripheral arterial disease. The incidence of hypertension is determined by 24-h ambulatory blood pressure monitoring. The non-cardiovascular complications occurring post HTx, including infections necessitating hospitalization, lymphoproliferative disease, other malignancies, renal dysfunction, etc. are available from the hospital records.

Acute cellular rejection was graded according to the 1990 recommendations of the International Society of Heart Lung Transplantation [22] updated in 2005 [23]. Rejection grades include none, mild (1990 grades 1A, 1B and 2), moderate (1990 grade 3A), and severe (1990 grades 3B and 4). If clinical features suggestive of acute antibody mediated rejection are present, the diagnosis was confirmed by light microscopy of EMBs until 2000. Thereafter HLA antibodies (LUMINEX, ThermoFisher Scientific, Pittsburg, PA) were determined and the diagnosis was confirmed by immunofluorescence or immunoperoxidase staining as recommended [23]. Cardiac allograft vasculopathy was graded into non-significant, mild, moderate and severe according to the 2010 nomenclature (Table 1) [21]. Right heart hemodynamic measurements were collected concurrently with each EMB procedure. We applied commonly used thresholds to mean atrial pressure (>5 mm Hg), mean pulmonary arterial pressure (>19 mm Hg) and pulmonary capillary wedge pressure (>12 mmHg) [24], which at rest in the supine position are consistent irrespective of sex and age [25,26].

**Table 1:** Nomenclature for cardiac allograft vasculopathy.

**Not significant:** no detectable angiographic lesions;
**Mild:** Angiographic left main (LM) <50%, or primary vessel (PM) with maximum lesion of <70%, or any branch stenosis <70% (including diffuse narrowing) without allograft dysfunction;
**Moderate:** Angiographic LM <50%, a PM ≥70%, or isolated branch stenosis ≥70% in branches of two systems, without allograft dysfunction;
**Severe:** Angiographic LM ≥50%, or two or more PMs ≥70% stenosis, or isolated branch stenosis ≥70% in all three systems, or mild to moderate lesions with allograft dysfunction (defined as LV ejection fraction ≤45% usually in the presence of regional wall motion abnormalities) or evidence of significant restrictive physiology.
**Definitions**
**Primary vessel** (PM) denotes the proximal and middle third of the left anterior descending artery, the left circumflex, the ramus and the dominant or co-dominant right coronary artery with the posterior descending and posterolateral branches.
**Primary vessel** (PM) denotes the proximal and middle third of the left anterior descending artery, the left circumflex, the ramus and the dominant or co-dominant right coronary artery with the posterior descending and posterolateral branches.
**Secondary branch vessels** include the distal third of the primary vessels or any segment within a large septal perforator, diagonals and obtuse marginal branches or any portion of a non-dominant right coronary artery.
**Restrictive cardiac allograft dysfunction** is symptomatic heart failure with echocardiographic E to A velocity ratio >2, shortened isovolumetric relaxation time (<60 msec), shortened deceleration time (<150 msec), or restrictive hemodynamic values (right atrial pressure >12 mm Hg, pulmonary capillary wedge pressure >25 mmHg, cardiac Index <2 l/min/m^2^).

Reproduced with permission from reference [21].

### Proteomics

#### Urinary proteomics

Under physiological conditions, about 70% of the urinary proteome originates from the kidney and the urinary tract, while 30% is derived from plasma [27]. Approximately 60% of the total mass of urinary peptides and proteins consist of collagen fragments [28]. A major advantage of UP profiling is the comfort for the patient, because all that is needed is a fresh mid-morning urine sample of 5 mL. Urinary proteins remain stable for a time long enough to perform UP profiling reliably [29]. Indeed, the UP does not undergo significant changes when urine is stored for 3 days at 4°C [30] or for 6 hours at room temperature [31]. Moreover, for studies running over a long time period, urine can be stored for years at -20°C without alteration of the proteome [29]. The urinary proteome is fairly well characterized and reference standards are available [32]. Detailed information on urine sample preparation, proteome analysis by CE-MS, data processing and sequencing of the urinary peptides allowing the identification of parent proteins is beyond the scope of this article, but is available in previous publications [32,33] and in the Data supplement (S1 File).

#### Tissue proteomics

The volume of EMBs is too small to allow TP studies. In a separate study, larger biopsies will be taken from explanted diseased hearts and disregarded donor hearts. In this substudy, the UP classifiers will be validated by demonstrating analogy between the proteomic profiles in urine and tissue samples of explanted hearts and differential expression of these proteins between diseased explanted hearts and unused donor hearts. Hence, urinary and tissue proteomics will help decipher complex molecular mechanisms related to HF and provide ways to improve its management. Access to comprehensive proteomics data combined with bioinformatics, will further enable the identification of drug targets as recently demonstrated in a study focusing on bladder cancer and showing the feasibility of this innovative approach, which combines TP with bioinformatics to identify drug targets [34]. As described in previous publications [35,36] and in the Data supplement (S1 File), tissue was digested with trypsin and the resulting peptides were analyzed using high-resolution LC-MS/MS (liquid chromatography coupled to tandem mass spectrometry). Protein identification was performed using the SEQUEST search engine (Proteome Discoverer 1.4, Thermo Scientific) against the SwissProt human protein database (30 May 2016), containing 20,197 entries without protein isoforms. Obtained results were further processed by applying the following filters: (i) high, medium and low confidence peptides; (ii) peptide rank up to 5; (iii) peptide grouping enabled or disabled. The list of peptides was exported from Proteome Discoverer and processed [37].

### Statistical analysis

SAS software (SAS institute, Cary, NC USA) version 9.4 was used for database management and statistical analysis. The central tendency and spread of the data are reported as mean and standard deviation. Departure from normality was evaluated by Shapiro–Wilk’s statistic and skewness (lack of symmetry) and kurtosis (tailedness) by the computation of the third and fourth moment about the mean divided by the cube of the standard deviation. The normal distribution was used to determine the significance of these distributional characteristics [38]. We compared means and proportions using Student’s t-test or ANOVA and the χ2 statistic, respectively. Statistical significance was an α-level less than 0.05 on two-sided tests. The statistical strategy for future analyses required to answer the research questions is summarized in the Data supplement (S1 File). In particular, how classifiers will be optimized for diagnostic accuracy in HTx patients is described in the Data supplement (S1 File, pages 11–12).

## Results

### Patient characteristics

At the time of writing of this article, the uPROPHET database included 352 recipients of a heart transplant. The underlying cause of treatment-resistant end-stage HF, which constituted the indication for HTx, was ischemic cardiomyopathy in 137 patients (38.9%), dilated cardiomyopathy in 142 (40.3%) and hypertrophic cardiomyopathy in 20 (5.7%). Among the remaining 53 patients, the etiology of HF included restrictive (n = 6), valvular (n = 13) or congenital (n = 23) heart disease, arrhythmogenic right ventricular dysplasia (n = 3), constrictive pericarditis (n = 1), cardiac sarcoidosis (n = 1), giant cell myocarditis (n = 2), primary pulmonary hypertension (n = 1), myocarditis (n = 2), and irreversible myocardial damage caused by a massive hemorrhage after rupture of the splenic artery (n = 1). The 352 patients underwent surgery in the interval ranging from August 1988 until April 2015 and included 86 women (24.4%). Mean (±SD) age of the donors was 35.8±13.4 years.

Table 2 lists the characteristics of the patients by etiology of HF at the time of collection of the first urine sample. Compared with dilated cardiomyopathy, patients with ischemic cardiomyopathy were older and more obese, had higher systolic and diastolic blood pressure, higher plasma glucose, serum creatinine, and lower estimated glomerular filtration rate (p≤0.016 for all). Diabetes mellitus and smoking were more prevalent in patients with ischemic compared with dilated cardiomyopathy (p<0.001). Table 3 list the characteristics of the patients enrolled in the study of TP at the time of writing of this manuscript.

**Table 2:** Patient characteristics by type of cardiomyopathy at first urine sampling.

Characteristic	Ischemic	Dilated	Hypertrophic	Other
Number	137	142	20	53
Frequency (%) of characteristic				
Women	22 (16.1)	35 (24.7)	6 (30.0)	23 (43.4)
Smokers	121 (88.3)	73 (51.4)	11 (55.0)	12 (22.6)
Hypertension	125 (91.2)	122 (85.9)	15 (75.0)	43 (81.1)
Diabetes mellitus	49 (35.8)	28 (19.7)	2 (10.0)	7 (13.2)
Mean (±SD) of characteristic				
Age (years)	63.5±9.61	54.1±16.2	49.6±18.4	47.4±17.0
Body mass index (kg/m^2^)	26.6±4.22	24.8±4.38	23.8±3.90	23.5±3.63
Donor age (years)	36.9±13.2	35.8±13.2	29.7±14.3	35.0±14.0
Office blood pressure				
Systolic (mm Hg)	148.8±22.8	138.1±18.8	136.6±19.7	138.8±20.5
Diastolic (mm Hg)	86.7±11.5	83.8±11.9	81.6±8.19	83.7±11.3
24-h ambulatory blood pressure				
Systolic (mm Hg)	126.7±12.2	123.7±11.8	121.1±11.3	120.2±9.2
Diastolic (mm Hg)	81.2±8.5	79.4±8.6	77.3±9.7	78.3±8.9
Biochemistry				
Serum creatinine (mg/dL)	1.57±0.50	1.39±0.55	1.29±0.41	1.29±0.40
eGFR (mL/min/1.73 m^2^)	49.9±18.7	63.0±28.4	68.1±29.5	66.0±25.2
Troponin T, μg/L	0.02±0.02	0.03±0.06	0.01±0.02	0.02±0.02
γ-GT (units/L)	33.9±33.6	34.1±34.3	39.3±34.9	48.9±65.7
Total cholesterol (mg/dL)	155.2±32.4	157.0±37.0	158.3±38.7	153.7±33.5
HDL cholesterol (mg/dL)	55.6±18.1	57.9±16.2	59.0±17.4	58.2±14.3
Plasma glucose (mg/dL)	107.2±27.0	99.4±26.3	90.9±15.7	93.4±10.2

Abbreviations: eGFR, estimated glomerular filtration rate derived from serum creatinine by the Chronic Kidney Disease Epidemiology Collaboration equation; γ-GT, gamma-glutamyltransferase; HDL, high-density lipoprotein. Other includes restrictive (n = 6), valvular (13) or congenital (23) heart disease, arrhythmogenic right ventricular dysplasia (3), constrictive pericarditis (1), cardiac sarcoidosis (1), giant cell myocarditis (2), primary pulmonary hypertension (1), myocarditis (2), and irreversible myocardial damage caused by a massive hemorrhage (1). 24-h ambulatory blood pressure was available in 133, 136, 20 and 49 patients with ischemic, dilated, hypertrophic or other cardiomyopathy, respectively.

**Table 3:** Characteristics of cases and controls.

Characteristic	Controls	Cardiomyopathy		
		Ischemic	Dilated	Hypertrophic
Number in category	7	15	14	3
Number of patients (%)				
Women	4 (57.1)	2 (12.3)	0	2 (40.0)
Hypertension	4 (80.0)	1 (8.3)*	2 (20.0)	2 (40.0)
Diabetes mellitus	0	0	1 (8.3)	0
Mean (±SD) of characteristic				
Age (years)	64.3±13.9	57.5±11.2	53.9±13.3	46.0±18.0
Body mass index (kg/m^2^)	24.8±3.7	26.3±4.4	25.9±3.7	22.3±2.6
Blood pressure (mm Hg)				
Systolic	143.2±31.9	126.9±14.7	106.3±11.6	105.4±21.5
Diastolic	75.8±34.8	76.6±9.9	69.3±13.0	70.6±10.1
Biochemical data				
Serum creatinine (μmol/L)	94.9±67.6	120.9±37.5	121.0±37.9	86.8±23.5
eGFR (mL/min/1.73 m^2^) [53]	73.5±32.3	60.5±22.1	63.5±23.3	87.7±26.4

eGFR indicates estimated glomerular filtration rate derived by the Chronic Kidney Disease Epidemiology Collaboration equation formula. Controls refers to normal donor hearts that were disregarded for transplantation. Hypertension and diabetes mellitus as documented in the medical records of cases and controls. In controls, blood pressure was the in-hospital blood pressure before removal of the donor heart and in cases the 24-h ambulatory blood pressure recorded within 3 months before transplantation.

Significance of the difference with controls: * p ≤ 0.05.

### Rejection status and anti-rejection drugs in UP patients

The median interval between HTx and the collection of the first urine sample in all 352 patients was 7.8 years (interquartile range, 3.3–14.3 years). In 13 patients, the HTx operation included in the uPROPHET database, was a retransplantation following the first HTx at a median interval of 11 years (interquartile range, 6.2–17.3 years). Table 4 summarizes the use of anti-rejection drugs by type of cardiomyopathy. Overall, at the time of the first urine collection, 79 (22.4%) patients were taking cyclosporine, 264 (75.0%) tacrolimus, 24 (6.8%) everolimus, 261 (74.2%) mofetil mycophenolate and 118 (33.5%) methylprednisolone. No patient was taking azothioprine. Of 352 patients, 41 (11.7%) were taking a single drug, 228 (64.8%) two drugs and 83 (23.6%) three drugs. The most common combination was tacrolimus plus mofetil mycophenolate, which was being used by 201 patients (57.1%).

**Table 4:** Medication use by type of cardiomyopathy.

Characteristic	Ischemic	Dilated	Hypertrophic	Other
Number in category	137	142	20	53
Anti-rejection drugs				
Cyclosporine	39 (28.5)	30 (21.1)	3 (15.0)	7 (13.2)
Tacrolimus	96 (70.1)	105 (73.9)	17 (85.0)	46 (86.8)
Everolimus	8 (5.8)	15 (10.6)	1 (5.0)	0 (0)
Mycophenolate	102 (74.5)	107 (75.4)	12 (60.0)	40 (75.5)
Methylprednisolone	46 (33.6)	50 (35.2)	4 (20.0)	18 (34.0)
Antihypertensive drugs				
Thiazide diuretics	19 (13.9)	11 (7.75)	0 (0)	4 (7.55)
Loop diuretics	15 (11.0)	7 (4.93)	4 (20.0)	3 (5.66)
β-blockers	56 (40.9)	51 (35.9)	5 (25.0)	11 (20.8)
ACEIs	53 (38.7)	54 (38.0)	6 (30.0)	14 (26.4)
ARBs	20 (14.6)	13 (9.15)	2 (10.0)	5 (9.43)
Calcium-channel blockers	47 (34.3)	37 (26.1)	6 (30.0)	16 (30.2)
Aldosterone antagonists	6 (4.38)	4 (2.82)	4 (20.0)	3 (5.66)
Centrally acting drugs	7 (5.11)	4 (2.82)	0 (0)	0 (0)
Antidiabetic drugs				
Oral drugs	27 (19.7)	20 (14.1)	1 (5.0)	6 (11.3)
Insulin	26 (19.0)	8 (5.63)	1 (5.0)	1 (1.89)

Values are number of patients (%). Abbreviations: ACEIs, angiotensin-converting enzyme inhibitors; ARBs, angiotensin type-1 receptor blockers.

The number of patients that underwent EMB within 6 months of the first urine sample totaled 79. Of these, 56 (70.9%) were in grade 0, 20 (25.3%) in grade 1A and 3 (3.8%) in grade 1B. Of 352 patients, 131 (37.2%) and 23 (6.5%) had a previous history of mild (grade = 1B) or severe (grade ≥ 2) acute cellular rejection. Acute anti-body mediated rejection had occurred in 22 (6.2%) patients. The number of follow-up urine samples available for analysis currently totaled over 950.

### Use of other drugs

At the time of the first urine collection, 60 (17.1%) patients were taking thiazide or loop diuretics, 123 (34.9%) β-blockers, 167 (47.4%) angiotensin-converting enzyme inhibitors or angiotensin I receptor blockers, 106 (30.1%) calcium-channel blockers and 17 (4.8%) aldosterone antagonists. The number of patients on antidiabetic treatment totaled 90, of whom 54 (15.3%) were on oral therapy and 36 (10.2%) on insulin.

### Baseline distributions of urinary proteomic classifier

**Fig 2:**
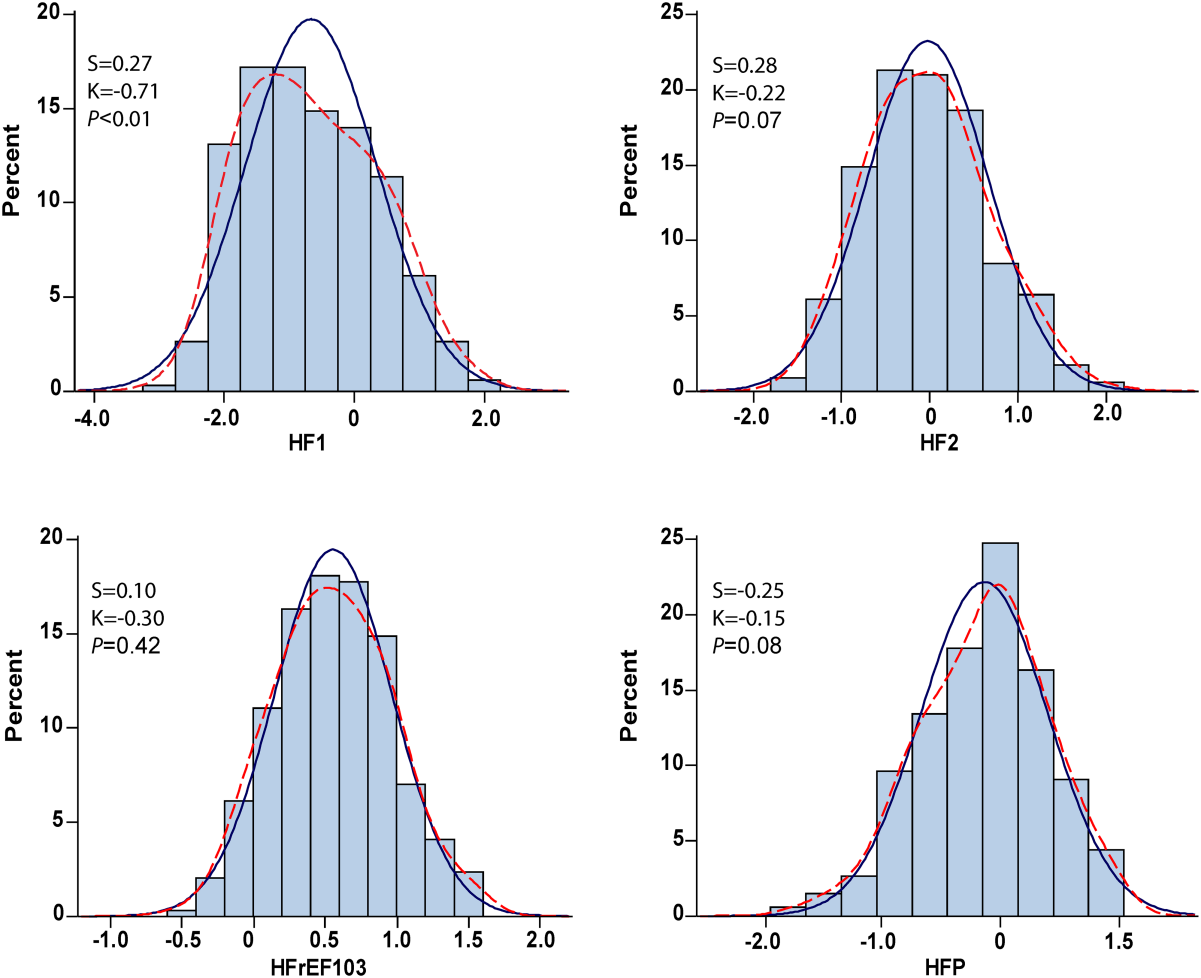
Distributions of the multidimensional urinary proteomic classifiers HF1, HF2, HfrEF103 and HFP, consisting of 85, 671, 103 and 96 urinary peptide fragments and designed for diagnosis and prognostication in the framework of subclinical diastolic left ventricular dysfunction (HF1) or symptomatic heart failure (HF2, HFrEF103 and HFP). The *P* value is for departure of the actually observed distribution (kernel distribution; dotted line) from normality (full line).

**Fig 3:**
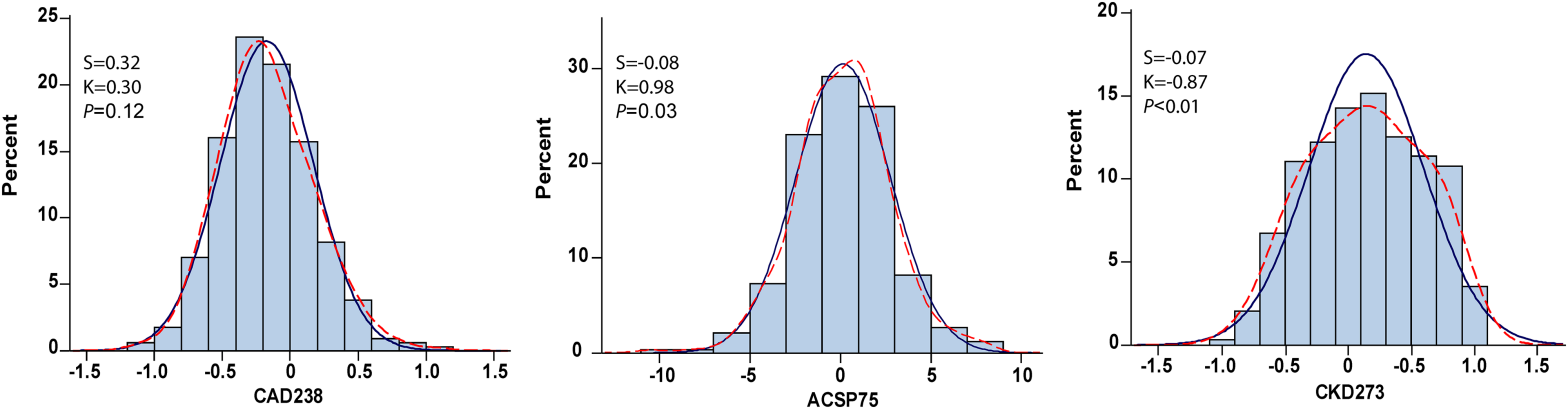
Distributions of the multidimensional urinary proteomic classifiers CAD238, ACSP75 and CKD273, consisting of 238, 75 and 273 urinary peptide fragments and designed for diagnosis and prognostication in the framework coronary heart disease (CAD238 and ACSP75) or chronic kidney disease (CKD273). The *P* value is for departure of the actually observed distribution (kernel distribution; dotted line) from normality (full line).

HF1 [5–7], HF2 [6], HFrEF103 [39], and HFP [40] are multidimensional UP classifiers, respectively consisting of 85, 671, 103 and 96 urinary peptide fragments, which were developed for diagnosis and risk stratification in the framework of subclinical diastolic left ventricular dysfunction (HF1) or symptomatic heart failure (HF2, HFrEF103 and HFP). CAD238 [11] and ACSP75 [11], consisting of 238 and 75 peptide fragments have been designed for diagnostic and prognostic use in coronary heart disease. CKD273 [8,9] consisting of 273 fragments is used for the early diagnosis and risk stratification in chronic kidney disease. Figs 2 and 3 show the distributions of the heart failure classifiers and of the coronary and chronic kidney disease classifiers respectively. The distributions of the UP biomarkers were normal with the exception of HF1 (Fig 2) and ACSP75 and CKD273 (Fig 3).

### Hemodynamic measurements at baseline

Table 5 lists the hemodynamic measurements obtained by echocardiography and right heart catheterization at the time of the first urine sampling. Between-group differences according to type of cardiomyopathy were mostly small and only reached significance for intraventricular septal thickness (12.4 *vs*. 11.5 mm; *P* = 0.0004), mean right atrial pressure (9.33 *vs*. 8.44 mm Hg; *P* = 0.010) and mean pulmonary artery pressure (22.9 *vs*. 20.9 mm Hg; *P* = 0.0004), which compared to the other groups were greater in patients with a history of ischemic cardiomyopathy, whereas their cardiac output was lower (4.82 *vs*. 5.17 L/min; *P* = 0.007).

**Table 5:** Hemodynamic measurements by type of cardiomyopathy.

Characteristic	Ischemic	Dilated	Hypertrophic	Other
Echocardiography				
Number	134	139	20	51
LV ejection fraction (%)	59.2±2.9	59.2±2.5	59.5±1.5	59.5±2.9
IVS thickness (mm)	12.4±2.4	11.6±2.2	11.7±2.4	11.2±2.3
LV end-diastolic diameter (mm)	41.7±5.8	42.2±5.3	44.1±6.0	41.6±4.5
E/A ratio	2.11±1.27	2.29±1.68	2.29±1.14	2.11±0.89
E/e' ratio	6.41±2.32	6.72±2.59	5.90±2.34	6.36±2.19
Right heart hemodynamics				
Number	123	120	19	44
Heart rate (bpm)	77.9±11.0	79.2±12.5	79.2±17.5	80.8±10.4
Cardiac output (L/min)	4.82±1.01	5.13±1.14	5.54±1.69	5.13±1.06
Mean RA pressure (mm Hg)	9.33±2.81	8.58±3.14	7.79±2.70	8.36±2.82
RV systolic pressure (mm Hg)	34.1±7.03	32.2±5.47	32.3±5.21	32.7±5.62
RV diastolic pressure (mm Hg)	9.55±3.25	9.10±3.41	8.05±2.70	8.77±3.58
Mean PA pressure (mm Hg)	22.9±5.1	20.9±4.4	20.4±3.6	21.0±4.5
Mean PCWP (mm Hg)	14.9±4.3	14.1±4.1	12.9±3.5	14.1±4.4

Values are mean±SD. Abbreviations: LV, left ventricle; IVS, interventricular septum; E, peak velocity of the transmitral blood flow during early diastole; A, peak velocity of the transmitral blood flow during late diastole; e’, peak velocity of the mitral annular movement during early diastole; RA, right atrium; RV, right ventricle; PA, pulmonary artery; PCWP, pulmonary capillary wedge pressure.

## Discussion

uPROPHET has the ambition to lay the foundation for the clinical application of UP in risk stratification in HTx patients and in monitoring the activity of the immune system and graft performance after HTx and to improve the management of HF in general. The research goals include: (i) construction of multidimensional UP classifiers that after HTx can help in detecting graft vasculopathy and monitoring the activity of the immune system and graft performance and in fine-tuning immunosuppression; (ii) sequencing of UP peptide fragments to identify key proteins mediating HTx-related complications; (iii) the validation of classifiers in a separate substudy by demonstrating analogy between the proteomic profiles in urine and diseased explanted hearts and by highlighting differences in the myocardial proteomic profiles between diseased explanted hearts and normal donor hearts not suitable for implantation; (iv) and the identification of novel drug targets. Although at the time of writing of this manuscript the analogy between the UP and TP studies seemed straightforward, the similarities between heart and urinary proteomic profiles might be more difficult to be picked up than anticipated. Indeed proteins and peptides might be modified in the circulating blood or in the kidney, particularly in the proximal tubule. On the other hand, we demonstrated that 298 transplanted patients, each characterized by one plasma and urine sample, were sufficient to demonstrate that right heart pressures were significantly correlated in multivariable adjusted analyses with plasma highly sensitive troponin T (hsTNT) and HF2.

According to a Milliman report (http://www.milliman.com/uploadedFiles/insight/Research/health-rr/1938HDP_20141230.pdf), the costs of HTx over a period from 3 months before surgery to six months after the intervention average approximately 1.25 million USD. Similar information about HTx costs in Europe is currently not available. However, expenditure in Europe is likely to be of the same order of magnitude as in the United States. Furthermore, EMB, the standard procedure to monitor rejection after HTx, comes at a cost of approximately 800€ per procedure in Belgium. Apart from the cost issue and the burden associated with an invasive procedure, EMB has additional limitations. The inter-observer variability in conclusively grading small tissue samples taken from the septal wall of the right ventricle is far from optimal [41]. The small EMB samples are not truly representative. Lesions indicative of rejection are patchy. Consequently, ambiguity hampers the histological cues as to whether a stage indicative of cellular rejection has been reached requiring adjustment of immunosuppression. Furthermore, in case of acute antibody-mediated rejection, cardiac biopsies are less informative [42]. This condition, characterized by myocardial capillary injury with endothelial cell swelling and intravascular macrophage accumulation, occurs in the first year in up to 15% of HTx patients [21]. The clinical presentation has no pathognomonic features, but the risk of graft failure is high, in particular in allosensitised recipients [21,42]. Another issue unaddressed by current state-of-the-art procedures is a reliable guide to taper or stop corticosteroids or reduce the dose of immunosuppressants in patients who survive the first year after HTx without major rejection.

The aforementioned issues highlight that the HTx field not only needs markers of rejection, but also markers that reflect the activity of the immune system. Increasing activity of the immune system predicts imminent rejection, expectedly months before rejection can be histologically confirmed. Conversely, declining activity of the immune system might signify that tapering of immunosuppression is possible. Pilot studies in recipients of kidney [43], liver [28] or bone marrow [44] allografts revealed unique UP signatures indicative of impending rejecting, including markers reflecting the activity of the immune system and inflammation [43,44]. These observations illustrate the feasibility of the ideas underlying this proof-of-concept project. uPROPHET will potentially break new grounds by proposing specific UP signatures that change in parallel with activation of the immune system and inflammation and that might guide immunosuppressant treatment in a way analogous to the use of the international normalized ratio (INR) in titrating vitamin K antagonists. In this way, the UP signature will complement the measurement of serum levels of immunosuppressive agents, which do not capture the true adherence of patients between successive blood samples.

A literature search, using as key words “rejection” AND “monitoring” AND “heart” AND “transplantation” did not reveal any competing ideas with the exception of the AlloMap^®^ test. This test assesses the gene expression profile, using RNA isolated from peripheral blood mononuclear cells amplified by a standard quantitative real-time polymerase chain reaction. A proof-of-concept clinical trial in the United States (CARGO) involved 602 selected patients who had received a cardiac transplant from 6 months to 5 years previously and who were at a low risk of rejection [45]. A randomized strategy of monitoring for rejection that involved gene-expression profiling, as compared with routine biopsies, was not associated with an increased risk of serious adverse outcomes and resulted in significantly fewer biopsies. In the United States, AlloMap^®^ testing has been carried out in over 80,000 blood samples (http://www.allomap.com/). The test is not used in Europe for various reasons. It is not validated for use within the first 6 months post HTx, the time window with the highest risk of rejection. Only one laboratory, located in California, offers the service. The cost per sample is as high as 2,000 USD. Because of the low positive predictive value, a positive test does not indicate rejection and has always to be confirmed by EMB.

uPROPHET builds on previously identified biomarkers and classifiers [5–9,11,39,40]. HF1 was originally designed in a case-control study nested within the Flemish Study on Environment, Genes and Health Outcomes (FLEMENGHO) [5] with the goal to identify potentially discriminating urinary biomarkers for diastolic LV dysfunction. To generate the HF2 classifier, all urinary proteomic datasets from HF cases available in the Mosaiques-Diagnostic database [28] were combined and compared with data from sex- and age-matched controls. Comparing cases with controls identified 671 potential biomarkers. The classifier had 88.7% accuracy, 87.8% sensitivity, and 89.6% specificity [6]. By design, HF1 is associated with early asymptomatic LV dysfunction [5], whereas HF2 reflects more advanced disease. In a proof-of-concept analysis of 745 FLEMENGHO participants [6], HF1 was associated with 0.204 cm/s lower e' peak velocity (*P* = 0.007) and 0.145 higher E/e' ratio (*P* = 0.020), while HF2 was associated with a 0.174 higher E/e' ratio (*P* = 0.008). According to published definitions [46,47], 67 (9.0%) participants had impaired LV relaxation and 96 (12.9%) had an elevated LV filling pressure. The odds of impaired relaxation associated with HF1 was 1.38 (*P* = 0.043) and that of increased LV filling pressure associated with HF2 was 1.38 (*P* = 0.052). HFP was originally designed in the Generation Scotland study and was subsequently validated in a cohort of FLEMENGHO participants with diastolic LV dysfunction at risk of HF [40]. HFrEF103 accurately (area under the curve, 0.972) discriminated between HF patients with reduced ejection fraction and control individuals with or without impaired renal function and hypertension (sensitivity, 93.6%) [39]. CAD238 and ACSP75 [11], are predictors of acute coronary events. CKD273 is a multidimensional urinary biomarker [48,49], enabling early detection of CKD and predicting deterioration of renal function [50,51]. The Food and Drug Administration (FDA) recently encouraged studies of CKD273 as a tool for early diagnosis and risk prediction in diabetic nephropathy [49]. Very recently, we demonstrated that CKD273 predicts progression to stage-3 CKD, defined as a decrease in estimated glomerular filtration by 10 mL/min/1.73 m2 or more to less than 60 mL/min/1.73 m2 over approximately 5 years [52]. In the initial stages of uPROPHET, these established urinary biomarkers [5–9,11,39,40] will be tested to detect worsening of right heart hemodynamic conditions (HF1 and HF2), coronary vasculopathy (CAD238 and ACSP75) or to predict decline in renal function (CKD273). Analyses of single sequenced urinary peptides, allowing to identify the parent proteins, will be pursued, as described elsewhere [7,10], as a way to gain insight in the underlying disease processes and molecular pathways.

Previous studies in the field of bladder cancer [36,37] highlight the feasibility of drug studies in the framework of uPROPHET. High resolution liquid chromatography coupled to tandem mass spectrometry analysis of tissue specimens from patients with non-muscle invasive (NMIBC, stage pTa) and muscle invasive bladder cancer (MIBC, stages pT2+) was conducted and identified 144 differentially expressed proteins between the two patient groups. Pathway and interactome analysis predicted strong activation in muscle invasive bladder cancer of pathways associated with protein synthesis e.g. eIF2 and mTOR signalling. Knock-down of eukaryotic translation initiation factor 3 subunit D (EIF3D) (overexpressed in muscle invasive disease) in metastatic T24M bladder cancer cells inhibited cell proliferation, migration, and colony formation in vitro and decreased tumor growth in xenograft models. Thereby, highlighting EIF3D as a potential therapeutic target in the previous study ensure the possibility of the identification of novel drug targets of the current project [37].

In conclusion, uPROPHET constitutes a vast resource for future urinary and tissue proteomic research in the field of HF and HTx. The evident benefit for the patients is the early and non-invasive detection of imminent complications, hence allowing a timely and targeted treatment approach. uPROPHET also aims, by analysis of single sequenced UP peptides and by searching for analogy between UP and myocardial proteomic signatures, to shed light on the pathophysiology of post-HTx complications. Combining uPROPHET findings with advanced bioinformatic tools might allow identifying novel molecular pathways and targets for intervention.

## Supporting information

S1 File Data supplement. (DOC)
