# Supplementary material for: Urinary Proteomics in Predicting Heart Transplantation Outcomes (uPROPHET)—Rationale and database description
Source: PLoS One. 2017 Sep 7;12(9):e0184443. doi: 10.1371/journal.pone.0184443 (PMC5589218; doi:10.1371/journal.pone.0184443)
Supplement: S1 File — (DOC) [file pone.0184443.s001.doc]

**PLOS ONE**

**Data supplement**

This Data Supplement formed part of the original submission and has been peer reviewed.
Supplement to “*Urinary Proteomics in Predicting Heart Transplantation Outcomes (uPROPHET) — Rationale and Database Construction*”

**Table of contents**

**Expanded methods** **p2**

Urinary proteomics **p2**

Tissue proteomics **p4**

Statistical methods **p7**

**References** **p14**

**S1 Table.** Schematic representation of statistical analysis **p19**

**S1 Fig.** Proteome coverage of 6 CE-MS runs of human urine standards **p20**

**S2 Fig.** Example of ‑log10 plot **p21**

**S3 Fig.** Example of V‑plot **p22**

**Expanded methods**

**Urinary proteomics**

Sample preparation and CE-MS analysis

For proteomic analysis, a 0.7 mL aliquot of stored urine was thawed immediately before use and diluted with 0.7 mL of 2 M urea, 10 mM NH4OH containing 0.02% sodium dodecyl sulphate. To remove higher molecular mass proteins, such as albumin and immunoglobulins, the sample was ultra-filtered, using Centrisart ultracentrifugation filter devices (20 kDa MWCO; Sartorius, Göttingen, Germany) at 3,000 relative centrifugal force units until 1.1 mL of filtrate was obtained. This filtrate was then applied onto a PD-10 desalting column (GE Healthcare, Uppsala, Sweden) equilibrated in 0.01% NH4OH in HPLC-grade in H2O (Carl Roth GmbH, Karlsruhe, Germany) to decrease matrix effects by removing urea, electrolytes, salts, and to enrich polypeptides. Finally, all samples were lyophilized, stored at 4°C, and suspended in HPLC-grade H2O shortly before CE-MS analyses.

CE-MS analyses were performed using a P/ACE MDQ capillary electrophoresis system (Beckman Coulter, Fullerton, CA) on-line coupled to a micrOTOF MS (Bruker Daltonics, Bremen, Germany) [1,2]. The electrospray ionization device (Agilent Technologies, Palo Alto, CA) was grounded, and the ion spray interface potential was set between –4 and –4.5 kV. Data acquisition and MS acquisition methods were automatically controlled by the CE via contact-close-relays. Spectra were accumulated every 3 seconds, over a mass-to-charge ratio (m/z) ranging from 350 to 3000.

Quality control

Accuracy, precision, selectivity, sensitivity, reproducibility (S1 Fig.) and stability of the CE-MS have been previously published [1,3]. Quality control involves daily CE-MS analysis of a human urine standard [3]. To prevent variability due to carry-over effects from one to the next analysis, capillaries are reconditioned between runs with 1 M NaOH. S1 Fig. shows the data from 6 CE-MS analyses of the human urine standard. The coefficient of variance estimated from over 600 human urine standard analyses for over 3 years was 5.8% [4].

Mass spectrometric data processing

Mass spectral peaks representing identical molecules at different charge states were deconvoluted into single masses, using MosaiquesVisu software [5]. Only signals with a charge >1 observed in a minimum of three consecutive spectra with a signal-to-noise ratio of at least 4 were considered. Reference signals of 1770 urinary polypeptides were used for CE-time calibration by locally weighted regression. For normalization of analytical and urine dilution variances, signal intensities were normalized relative to 29 ‘‘*housekeeping*’’ peptides [6,7]. The obtained peak lists characterize each polypeptide by its molecular mass, normalized CE migration time and normalized signal intensity. All detected peptides were deposited, matched, and annotated in a Microsoft SQL database, allowing further statistical analysis [8]. For clustering, peptides in different samples were considered identical, if mass deviation was less than 50 ppm. CE migration time was controlled to be below 0.35 minutes after calibration.

Sequencing of polypeptides

CE-MS signals were in silico assigned to the previously sequenced peptides from Human Urinary Proteome Database, version 2.0 [9]. Peptides from this database were sequenced, as described elsewhere [10,11]. Briefly, urinary peptides were fragmented, using different tandem mass spectrometric techniques with a prior separation step with CE or HPLC. Fragmentation spectra were matched to the protein sequences from up-to-date public databases (IPI, NCBI Reference Sequence Database and Uniprot), using MS/MS search engines MASCOT (Matrix Sciences Ltd., London, UK) and OMSSA (National Center for Biotechnology Information, Bethesda, MD). In matching, we accounted for urinary proteins post-translational modifications, such as hydroxylation of lysine and proline, and specific MS characteristics. Peptide sequences from LC-MS/MS analyses were verified by the comparison of experimental and theoretical CE migration time, which is dependent on the number of basic and neutral polar amino acids.

Identified specific urinary peptides were combined into multidimensional classifiers, using the support vector machine-based MosaCluster software, version 1.7.0 [12]. MosaCluster calculates classification scores based on the amplitudes of the selected biomarkers. Classification is performed by determining the Euclidian distance (defined as the support-vector machine classification score) of the vector to a maximal margin hyperplane.

**Tissue proteomics**

Processing of samples

Approximately 30 mg of heart tissue will be homogenized in 150 μL of lysis buffer (4% SDS, 0.1 M DTE, 0.1 M Tris-HCl pH 7.6), using a bullet blender homogenizer (Next Advance, Averill Park, NY). Samples are homogenized using stainless steel beads (0.9-2 mm diameter) at speed 12 during 5 minutes, followed by a second homogenization step at speed 10 for 3 minutes. Samples are then centrifuged at 16,000 g for 10 minutes at room temperature and the supernatant is transferred to clean tubes. Protein concentration is determined by the Bradford assay (BioRad, Hercules, CA). Subsequently, protein extracts (200 μg) are processed using the FASP method (Filter Aided Sample Preparation), as described previously [13-16].

Tryptic digests are analyzed by nano-flow liquid chromatography-tandem mass spectrometry (LC-MS/MS), using Orbitrap Velos Fourier Transform Ion Cyclotron Resonance Mass Spectrometer (FTMS) (Thermo Finnigan, Bremen, Germany), as described previously [14,17-19]. Lyophilized peptides are re-dissolved in 200 μL of HPLC grade water. Subsequently, 5 μL of the peptide mixture is analyzed on a nano-flow system (Dionex Ultimate 3000 RSLS, Dionex, Camberley UK). Briefly, samples are loaded onto a Dionex nano-trap column (C18; 0.1 ×  20 mm; 5 μm) at a flow rate of 5 μl/min in 98% 0.1% formic acid and 2% acetonitrile, followed by elution onto an Acclaim PepMap nano-column (C18; 75 μm × 50 cm; 2 μm; 100 Å) at a flow rate of 0.3 μL/min. Reverse-phase chromatography is performed using a linear gradient of solution A (0.1% formic acid and acetonitrile [98:2]) and solution B (0.1% formic acid and acetonitrile [20:80]). Separation is initiated using 1% solution B (5 minutes), followed by a gradual increase to 20% (360 minutes) and 45% (480 minutes). Ionization involves a nano-electrospray source (Proxeon, Thermo Fisher Hemel Hempstead, UK) in positive ion mode and mass spectral analysis is performed in an Orbitrap Velos FTMS. Ionization voltage is 2.6 kV and the capillary temperature is set at 250 °C. The mass spectrometer is operated in MS/MS mode scanning from 380–1,600 amu (atomic mass unit). The MS analysis is performed using a data-dependent acquisition (top 20). Resolution in MS1 is 60,000 and in MS2 7,500. Parent ions are fragmented at an energy setting of 40 by higher energy collision-induced dissociation.

Protein identification

As described in previous publications[15,16,19], protein identification is performed using the SEQUEST search engine (Proteome Discoverer 1.4, Thermo Scientific) against the SwissProt human protein database. Obtained results are further processed by applying the following filters: (i) high, medium and low confidence peptides; (ii) peptide rank up to 5; (iii) peptide grouping enabled or disabled. The list of peptides is exported from “*Proteome Discoverer*” and processed further as follows using in-house developed software, where these peptide sequences are harmonized, so that the most probable sequence per peptide is assigned, improving the data consistency.

Protein identification involves five steps. (i) Calibration: To re-identify the same peptide across the whole study by m/z and retention time (RT), the exported peptide list from one sample, which covers the full mass and retention time range is selected and all the remaining samples are aligned to this representative one. (ii) Clustering: we virtually draw all data points (pairs of m/z and RT) from all the proteome discoverer exports in a 2‑dimensional plane. Then, we take rectangles with fixed size (5 ppm for the mass and 15 minutes for the retention time are common) and moved them until they individually captured most of the data points. The center of these rectangles (pairs of m/z and RT) are used to establish a cluster list. This “*cluster list*” is considered the list of all peptides that can be found in all the samples of the study. (iii) Matching and Sequence Assignment: Each of the clusters from the cluster list has a unique ID. After matching the exported peptide lists from each sample to the cluster list, each line of each peptide list has an ID. For each cluster from the list, we determine the set of sequences that have the same ID; from that data set, the sequence with highest frequency across the study is chosen. If there is a tie, we select the one with the highest maximum validity score (Xcorr). The rectangles construed in step 2 can intersect; hence, in rare cases one sequence can be assigned to more than one cluster. (iv) Protein Annotation: each Uniprot ID is annotated with protein name and protein symbol, based on the uniprot database. In the case of one peptide being assigned to multiple protein accessions, the protein occurring more frequently across the cluster list is selected. (v) Retrieve Peptide Information: peptide area, confidence, and mass/RT information are retrieved from the exported peptide lists of each sample, which now have the cluster IDs. Only peptides with deviation <5 ppm between experimental and theoretical mass are retained at this step.

All new peptide lists of each sample are merged based on the cluster ID and duplicate sequences are combined by summing up their areas. For a limited number of sequences, for which no peptide area can be retrieved by Proteome Discoverer, the absent values are replaced with the mean area under the peak of that group. When the peptide is not identified in a particular sample, the missing value is set to zero. Only peptides reported in more than 60% of the samples in at least one experimental group (cases or controls) are considered for the calculations of the number of peptide and protein identification, protein peak areas, evaluation of consistency and statistical analysis. Subsequently, part per million (ppm)-normalization of the protein peak areas is conducted according to the following formula [18,20]: normalized peak area = (peptide peak area/total peak area) × 106. Protein abundance in each sample is calculated as the sum of all normalized peptide areas for a given protein.

**Workflow of future statistical analyses**

For database management and statistical analysis, we used the SAS system, version 9.4 (SAS Institute Inc., Cary, NC). The proposed statistical workflow is summarized in S1 Table.

Preparing for analysis

Before analysis, the distribution of continuous variables will be checked for deviation from normality. Outliers will be removed if an individual’s value is 3‑SDs or more distant from the group mean. If necessary, distributions of variables will be normalized by a logarithmic or other transformation. The distributions of the urinary peptides will be rank normalized by sorting measurements from the smallest to the highest and then applying the inverse cumulative normal function [21].

Basic statistical approaches

Before any adjustment or multivariable modelling, most analyses will start by showing patient characteristics by categories, such as quantiles of an exposure variable (a multidimensional UP classifier) or type of cardiomyopathy or by making scatterplots of outcome versus exposure variables. Means will be compared using the large-sample z‑test, t‑test or ANOVA, proportions by the  χ^2^ statistic or Fisher’s exact test, and survival function estimates by the log-rank test. If prevalence or incidence rates need standardization across subgroups, we will use the direct method. We will calculate 95% confidence intervals of rates as $R\pm\text{ }\sqrt{(R/T)}$, where R and T are the rate and the denominator used to calculate the rate. Significance will be a 2‑sided α‑level of 0.05 or less.

Identification of application of covariables

To identify covariables to be retained in the analyses, continuous outcomes or categorical outcomes will be regressed on covariables of potential relevance, using linear or logistic regression, using a stepwise procedure with *P*‑values for covariables to enter and stay in the model set at 0.15. This is the default in the SAS package and allows for retaining covariables in the statistical modelling that are not formally significant, but might still be relevant. Once a group of covariables is identified, a constant set will be used throughout a given analysis for all related continuous and categorical outcomes. For continuous outcomes, accounting for covariables will be done by standardization of the outcomes of interest, using the β-coefficients of the regression model used to identify the set of relevant covariables or by including the covariables and the exposure variable (e.g. the UP classifier) in the same regression model. Both approaches yield the same estimates for the associations between the outcomes and exposure variables, but standardization is often easier to implement in programming.

Analyses of continuous outcomes

The same principles apply to the cross-sectional and longitudinal analyses of a continuous outcome. While accounting for covariables, we will regress the continuous outcome of interest on the urinary peptide markers and construct –log10 probability plots (for example, see S2 Fig. [22]). Based on the number of UP markers, we will apply Bonferroni correction, Bonferroni step-down (Holm) correction or the Benjamini and Hochberg false discovery rate. In longitudinal analyses, in which a continuous outcome (e.g. right heart hemodynamic measurements) is predicted from a baseline biomarker, the baseline value of the trait of interest will be accounted for. If outcome, biomarkers and covariables are available at multiple time points, mixed models will be used to account for clustering of observations within patients. Mixed models can also accommodate randomly missing values or a variable number of time points per individual.

For analyses of single urinary peptides, which have been sequenced and which can identify the protein from which they are derived, we will select those peptides that have a detectable signal in at least 95% of participants. Ignoring biomarkers with missing values might waste potentially important information, explaining why in previous studies of a more exploratory nature, this threshold was relaxed to 70% [23] or even lower [24]. However, as a major objective of uPROPHET is to deepen insight in the pathophysiological pathways leading to adverse outcomes in HTx patients, we chose to apply a more stringent criterion in uPROPHET, thereby avoiding the possibility of false positive findings.

In analyses of multiple urinary peptides combined, we will apply partial least squares (PLS) analysis, which is a statistical technique that constructs models for continuous outcomes in relation to correlated high-dimensional explanatory variables [25]. PLS allows identifying a set of independent latent factors that are linear combinations of the urinary peptides and that maximize the covariance between the urinary peptides and the variables describing the outcome of interest. We will retain the smallest number of latent factors, for which the predicted residual sums of squares (PRESS, calculated using leave-one-out cross-validation) does not differ significantly (*P*>0.10) from the model with the minimum PRESS value, as assessed by the van der Voet T^2^ statistic. The importance of each urinary marker in the construction of the PLS factors will assessed from the Variable Importance in Projection (VIP) scores of Wold with the threshold set at approximately 1.5. PLS allows constructing V‑plots, in which VIP scores and the rescaled and centered correlation coefficient among variables are plotted along the vertical and horizontal axis, respectively (for example, see S3 Fig. [22]). Plotted biomarkers associated with high VIP score and low correlation coefficients (top left quadrant of the plot) identify predictors of an adverse outcome, whereas those associated with high VIP score but high correlation coefficient (top right quadrant) are inversely associated with an outcome. The PLS approach does not require to adjust for multiple testing.

Analyses of categorical outcomes

Analyses of categorical outcomes will follow the same principles as those with continuous outcomes. Multivariable-adjusted relative risk can be computed by logistic regression or proportional hazard (Cox) regression. Logistic regression is appropriate for cross-sectional designs or prospective analyses, in which the follow-up duration is approximately similar in all patients. To model time to an adverse health outcome or until the censoring date, Cox regression will be the approach of choice. Mixed models as implemented in the SAS software can also accommodate categorical outcomes. If multiple biomarkers are assessed simultaneously, then constructing –log10 plots provides a way to present the results graphically and to adjust for multiple testing. Partial least square discriminant analysis (PLS-DA) combines highly correlated biomarkers into a single analysis and allows constructing V-plots for categorical outcomes.

Evaluation of added diagnostic or predictive accuracy

If the discriminatory threshold of a biomarker is known, computing its diagnostic or predictive value can be simply done from 2-by-2 tables providing sensitivity, specificity, positive and negative predictive value and the misclassification rate. Running a published SAS macro [26] allows determining thresholds for a biomarker yielding a specificity of 90%. Optimal discrimination limits for a biomarker can be determined by maximizing the Younden index (the maximum of sensitivity plus specificity minus 1).

The added value of a biomarker (continuous or categorical), over and beyond a set of covariables, can be assessed from the integrated discrimination improvement (IDI) and the net reclassification improvement (NRI) [27,28]. IDI is the difference between the discrimination slopes of the basic model and the basic model extended with the biomarker. The discrimination slope is the difference in predicted probabilities (%) between subjects with and without endpoint. To calculate NRI, we predicted in each participant the risk of a renal event from a Cox model with and without the biomarker. If P(up/event) is the percentage of subjects with events whose predicted probability is increased by adding the biomarker to the model and if P(up/nonevent) is the percentage of subjects without events whose predicted probability is increased, then NRI equals 2 × (P[up/event] – P[up/nonevent]). IDI and NRI provide complementary information. Indeed, if adding a biomarker to a model increases the predicted probability in cases, this is reflected by a significant increase in IDI, while NRI indicates the extent by which a biomarker improves diagnostic accuracy. Although applied frequently, expert statisticians suggested that IDI and NRI have limitations [29]. They recommended retaining existing descriptive terms, such as the true-positive and false-positive classification rates, or testing the null hypothesis of no prediction increment from modelled regression coefficients.

Finally, the capability to discriminate between patients with or without an adverse health outcomes can also be assessed by constructing receiver operating characteristic (ROC) curves and by calculating the area under the ROC curve (AUC). The DeLong method provides a way to compute 95% confidence intervals of the AUC.

Molecular pathways

To ensure detection of relevant molecular pathways and to build a network of biologically meaningful interactions, advanced bioinformatics tools will be used in combination with the literature [19,30]. Functional analysis of the features will be performed using among others the open-source tools, such as the Protein Annotation Through Evolutionary Relationship (PANTHER) software [31] or DAVID bioinformatics [32]. Additional tools include Ingenuity Pathway Analysis (IPA) and Cytoscape’s plugins like ClueGO and CluePedia. In addition, proteases responsible for the generation of urinary biomarkers will be investigated in silico using Proteasix [33] and the information obtained in the pathway analysis. The hypothesis is that changes in protease activity are linked to disease pathophysiology.

**References**

**S1 Table.**

**Schematic representation of the work flow of future statistical analyses**

| **Analysis step** | **Statistical method** |
| --- | --- |
| Preparing for analysis | Checking distributions, logarithmic transformation, rank normalization, removal of outliers |
| Basic statistical approaches | Large-sample z test, t-test or ANOVA (means); χ2 statistic or Fisher exact test (proportion); log-rank test (survival functions); analyses across quantiles of biomarkers; scatterplots; standardization of rates |
| Identification of covariables | Stepwise linear or logistic regression |
| Analyses with continuous outcome |  |
| Single urinary peptides, one at a time |  |
| Cross-sectional analyses | Multivariable-adjusted linear regression, correction for multiple testing |
| Longitudinal analyses | Multivariable-adjusted linear regression (including adjustment for the baseline value of the outcome, if available), correction for multiple testing |
| All urinary peptides |  |
| Cross-sectional analyses | Partial least squares analysis |
| Longitudinal analyses | Partial least squares analysis |
| Analyses with categorical outcome |  |
| Single urinary peptides, one at a time |  |
| Cross-sectional analyses | Multivariable-adjusted logistic regression, correction for multiple testing |
| Longitudinal analyses | Multivariable-adjusted Cox regression, correction for multiple testing |
| All urinary peptides |  |
| Cross-sectional analyses | Partial least squares discriminant analysis |
| Longitudinal analyses | Partial least squares discriminant analysis |
| Prediction of adverse outcomes | Integrated discrimination improvement, net reclassification improvement, optimized thresholds, 2-by-2 classification tables, log-rank test, receiver operating characteristic curve, |
| Molecular pathways | PANTHER, DAVID, IPA, Cytoscape, Proteasix |


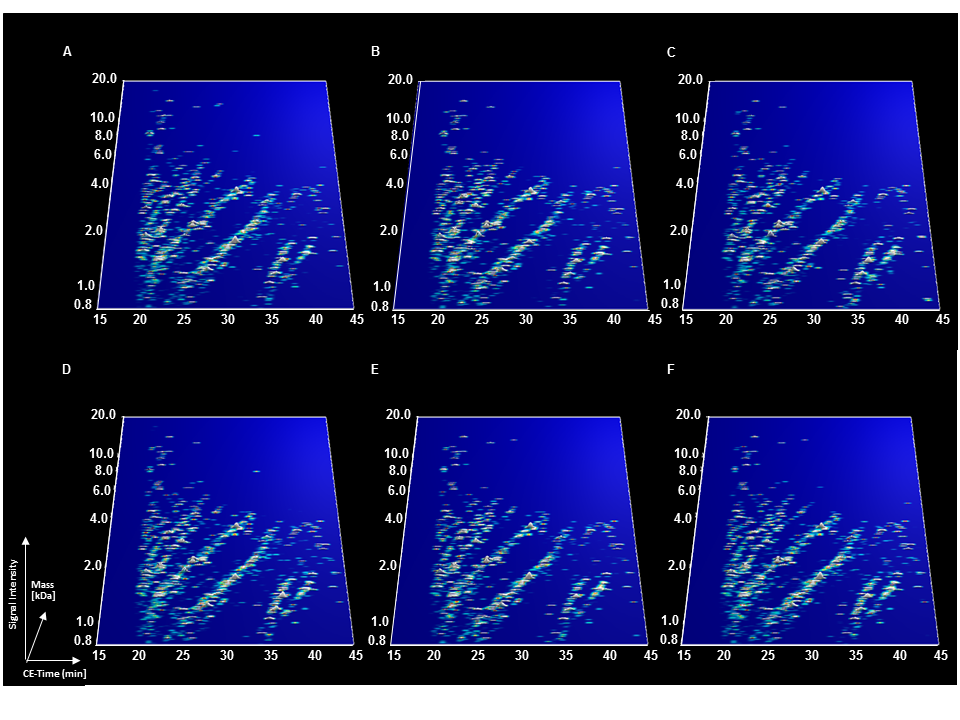


**S1 Fig.**

**Proteome coverage of 6 CE-MS runs (A–F) of human urine standards.**
The molecular mass on a logarithmic scale (0.8-20 kDa on the y-axis) was plotted against the normalized CE migration time (15-45 min on the x-axis). Peak height and color represent average signal intensity. The human urine standard is a urine sample from a randomly selected healthy person that is used for quality control (reference [3]).

**S2 Fig.**

**–Log10(p) probability plot of the multivariable-adjusted associations of renal function phenotypes with the urinary peptides.** eGFR indicates estimated glomerular filtration rate. All analyses were adjusted for mean arterial pressure, waist-to-hip ratio, smoking, plasma glucose, γ‑glutamyltransferase, total-to-HDL cholesterol ratio, 24‑h albuminuria, and use of diuretics, inhibitors of the renin-angiotensin system (β‑blockers, angiotensin-converting-enzyme inhibitors and angiotensin type‑1 receptor blockers) and vasodilators (calcium-channel blockers and α‑blockers). The longitudinal analysis of change in eGFR as continuous variable was additionally adjusted for baseline eGFR and follow-up duration. The horizontal line denotes the significance level with Bonferroni correction applied. Red dots represent mucin‑1 and green dots fragments of collagen I or III or fibrinogen. Reproduced from reference [22] under the CC BY-NC-ND license.


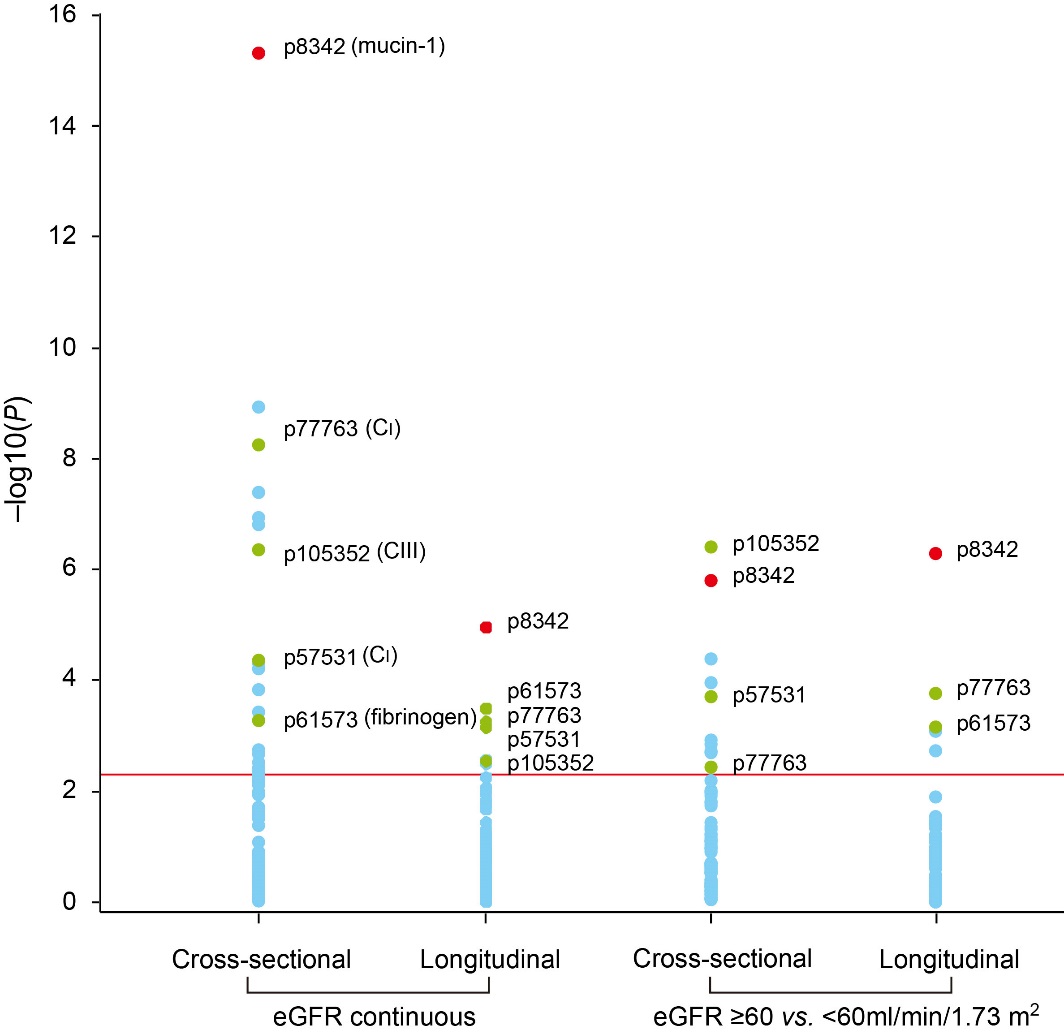


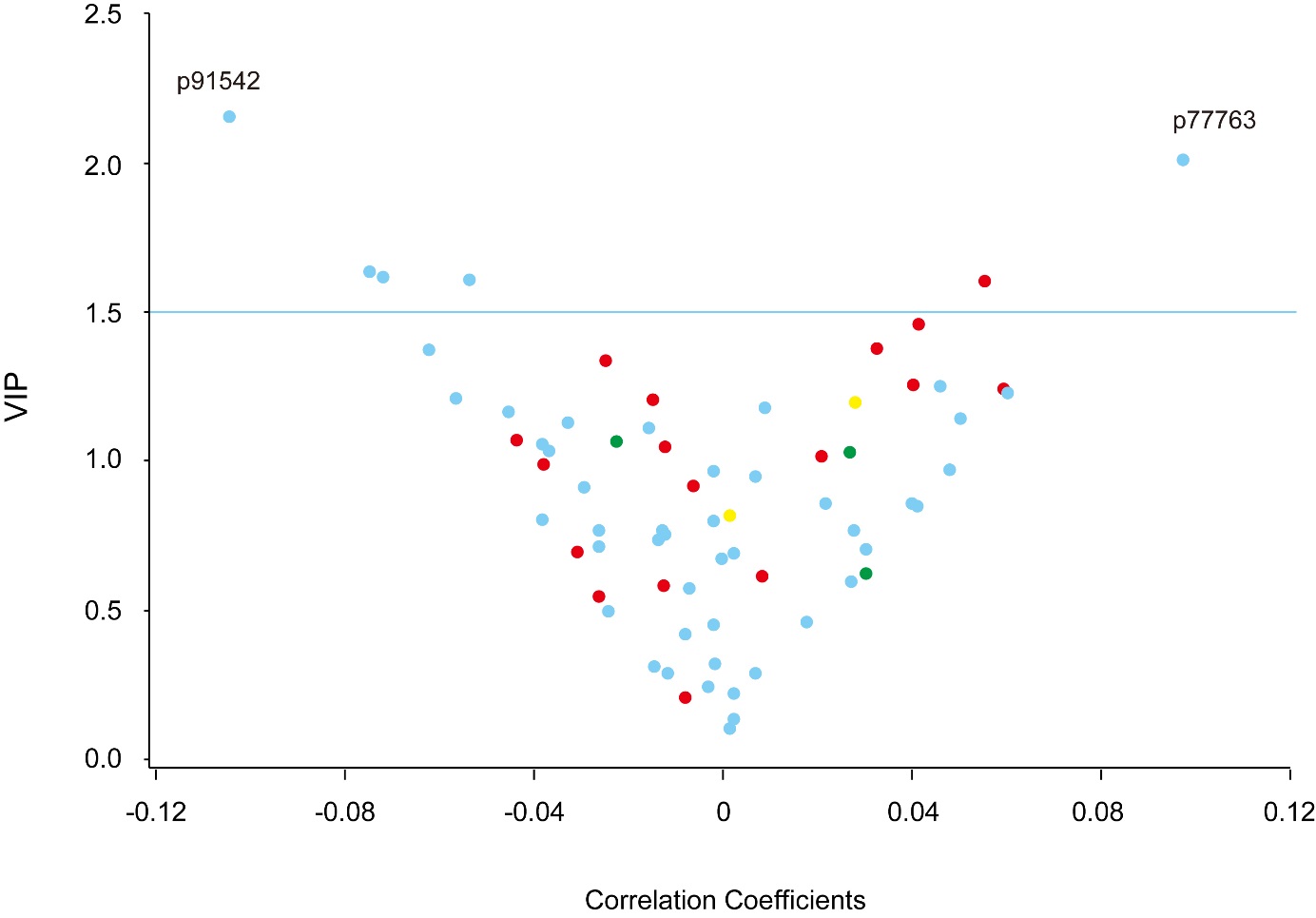


**S3 Fig.**

**V-plots generated by partial least square analysis.** Variable Importance in Projection (VIP) scores indicate the importance of each urinary fragment in the construction of the partial least square factors and are plotted against the centered and rescaled correlation coefficients. The correlation coefficients reflect the associations of the E/e’ with the sequenced urinary peptide fragments. Fragments associated with lower E/e’ (left side of the V‑plot) include, among others, p91542. p77763 was associated with higher E/e’ (right side of the V‑plot). Fragments derived from collagen I, III, IV and V are labelled blue, red, yellow and green, respectively.
